# Replicated Evidence of Absence of Association between Serum S100B and (Risk of) Psychotic Disorder

**DOI:** 10.1371/journal.pone.0082535

**Published:** 2013-12-17

**Authors:** Christine van der Leeuw, Machteld Marcelis, Sanne C. T. Peeters, Marcel M. Verbeek, Paul P. C. A. Menheere, Lieuwe de Haan, Jim van Os, Nico J. M. van Beveren, for Genetic Risk and Outcome in Psychosis (G.R.O.U.P.)

**Affiliations:** 1 Department of Psychiatry and Psychology, School for Mental Health and Neuroscience, European Graduate School of Neuroscience, Maastricht University Medical Centre, Maastricht, The Netherlands; 2 Departments of Neurology and Laboratory Medicine, Radboud University Medical Centre, Nijmegen, The Netherlands; 3 Donders Institute for Brain, Cognition and Behaviour, Nijmegen, The Netherlands; 4 Central Diagnostic Laboratory, Maastricht University Medical Centre, Maastricht, The Netherlands; 5 Deparment of Psychiatry, Academic Medical Centre, Amsterdam, The Netherlands; 6 King's College London, King's Health Partners, Department of Psychosis Studies, Institute of Psychiatry, London, United Kingdom; 7 Departments of Psychiatry and Neuroscience, Erasmus Medical Centre, Rotterdam, The Netherlands; 8 Department “Nieuwe Kennis”, Delta Centre for Mental Health Care, Rotterdam, The Netherlands; Cleveland Clnic Foundation, United States of America

## Abstract

**Background:**

S100B is a potential marker of neurological and psychiatric illness. In schizophrenia, increased S100B levels, as well as associations with acute positive and persisting negative symptoms, have been reported. It remains unclear whether S100B elevation, which possibly reflects glial dysfunction, is the consequence of disease or compensatory processes, or whether it is an indicator of familial risk.

**Methods:**

Serum samples were acquired from two large independent family samples (n = 348 and n = 254) in the Netherlands comprising patients with psychotic disorder (n = 140 and n = 82), non-psychotic siblings of patients with psychotic disorder (n = 125 and n = 94) and controls (n = 83 and n = 78). S100B was analyzed with a Liaison automated chemiluminescence system. Associations between familial risk of psychotic disorder and S100B were examined.

**Results:**

Results showed that S100B levels in patients (P) and siblings (S) were not significantly different from controls (C) (dataset 1: P vs. C: B = 0.004, 95% CI −0.005 to 0.013, p = 0.351; S vs. C: B = 0.000, 95% CI −0.009 to 0.008, p = 0.926; and dataset 2: P vs. C: B = 0.008, 95% CI −0.011 to 0.028, p = 0.410; S vs. C: B = 0.002, 95% CI −0.016 to 0.021, p = 0.797). In patients, negative symptoms were positively associated with S100B (B = 0.001, 95% CI 0.000 to 0.002, p = 0.005) in one of the datasets, however with failure of replication in the other. There was no significant association between S100B and positive symptoms or present use or type of antipsychotic medication.

**Conclusions:**

S100B is neither an intermediate phenotype, nor a trait marker for psychotic illness.

## Introduction

The search for biological markers and endophenotypes is ongoing in the field of psychiatry. The term biomarker refers to a characteristic that can be objectively measured and evaluated as an indicator of normal and pathogenic biological processes [1]. Their identification may offer insight into etiological mechanisms of disease and offer new treatment perspectives. In clinical practice, valid biomarkers assist in the diagnostic process and enable monitoring of disease course and treatment response [1], [2].

S100B is a protein that has been described as a potential biomarker of neurological and psychiatric disease. It has been coined “the CRP (C-reactive protein) of the brain” [3], and is an indicator of central nervous system (CNS) pathology from birth until elderly age [4]. S100B is expressed in and secreted by glial cells, with dual effects: at physiological intracellular levels and nanomolar extracellular concentrations it is neurotrophic [5], [6]; at micromolar levels it becomes neurotoxic [3], [5]–[7]. Elevated serum S100B in schizophrenia has been fairly consistently reported since the first publication more than a decade ago [8], and has been confirmed by a meta-analysis [9]. This meta-analysis, however, was not based on systematic review and did not report on apparently high levels of heterogeneity. Also, studies included were typically very small and no analysis of possible publication bias was provided. A few studies reported absence of association or inconclusive findings [10]–[12].

Initially, S100B elevation was ascribed to glial dysfunction or astrocytic activation [13], [14]. It is not understood whether it results from passive release secondary to neuronal damage, or active secretion due to glial activation as a primary pathophysiological or compensatory mechanism. Indirect evidence points to active secretion illustrated by reports of isolated elevation of S100B levels, unaccompanied by established markers of neuronal damage [9], [11]. In addition, increased S100B levels have been associated with increased metabolism in glial cells measured by magnetic resonance spectroscopy [15]. Clinically, elevated S100B may be associated with acute psychosis and persistent negative symptoms [10], [16]–[18]. Histological findings indicate that glial activation may result in increased intracellular S100B in paranoid schizophrenia (positive symptoms), whereas white matter damage or dysfunction in residual schizophrenia (negative symptoms) may lead to extracellular release [19].

More recently, S100B in schizophrenia has been related to glucose metabolism and insulin resistance [20]. Altered glucose metabolism in schizophrenia is frequently associated with the use of second-generation antipsychotics, but changes in insulin resistance and insulin-related peptides have also been observed in antipsychotic-naïve patients [21], [22], suggesting an etiologic role for hyperinsulinemia. The two mechanisms may not be mutually exclusive. Abnormal glucose metabolism (i.e. diabetes) leads to small vessel disease, which causes white matter lesions. Accordingly, glucose abnormalities may cause secondary glial dysfunction, with or without primary altered glial function.

Notably, both white matter irregularities [23]–[26] and glucose abnormalities [27]–[29] have been reported in unaffected first-degree relatives of patients with schizophrenia. However, it is not known whether S100B changes exist in this group. Two Chinese studies have investigated S100B gene polymorphisms as possible indicators of susceptibility to schizophrenia. The first study [30] found that the V3–V4 (G-C) haplotype of the S100B gene was more prevalent in patients than controls, whereas the second study [31] reported an absence of significant differences in genotypes or allele frequencies of S100B gene polymorphisms between patients and controls.

In summary, it remains unclear whether S100B elevation is associated with schizophrenia, and if so, whether the association reflects the consequence of acute or chronic disease processes, compensatory mechanisms or genetic risk for schizophrenia. We set out to test whether S100B levels are associated with (familial risk of) psychotic disorder, in two large and independent samples. We hypothesized that S100B levels would be elevated in both siblings and patients, suggesting that S100B elevation may be considered an intermediate phenotype.

## Materials and Methods

### Subjects

Data was collected in the context of an ongoing longitudinal multicentre study in the Netherlands [32], [33]. The present total study population comprised cross-sectional data from two large independent samples derived from: i) Amsterdam and Rotterdam, including surrounding areas, and ii) Maastricht and surrounding areas (extending into nearby Belgium) (hereafter referred to as the Amsterdam and Maastricht samples respectively). Patients with a minimum age of 16 years with a diagnosis of non-affective psychotic disorder were included. Patients were recruited through the mental health services where they were treated, either as in or outpatients. Diagnosis was based on DSM-IV criteria [34], assessed with the Comprehensive Assessment of Symptoms and History (CASH) interview [35]. Siblings were sampled through participating patients. On a few occasions, the patient refused participation but the sibling wished to participate, in which case the sibling was included independently. The CASH was also used to confirm the absence of a diagnosis of non-affective psychosis in the siblings, and absence of lifetime diagnosis of psychotic disorder in the control subjects. Control subjects were recruited using random mailings in nearby municipalities and through advertisement in newspapers. For the control subjects, the occurrence of any psychotic disorder in either the subject or any first-degree family member, assessed using the Family Interview for Genetic Studies, constituted an exclusion criterion.

Additional exclusion criteria constituted oncologic processes, autoimmune disease, current infectious disease, cardiovascular disease and neurological disease.

#### Amsterdam sample

The Amsterdam sample comprised 140 patients, 125 non-psychotic siblings and 83 controls. Patients in this sample were diagnosed with schizophrenia (n = 89), schizophreniform disorder (n = 6), schizoaffective disorder (n = 19), delusional disorder (n = 1), substance-induced psychotic disorder (n = 2), brief psychotic disorder (n = 1) and psychotic disorder NOS (n = 22). There were 10 siblings with a diagnosis of major depressive disorder (MDD), two siblings with bipolar disorder, two siblings with developmental disorders and one sibling with anorexia nervosa. Among the controls, 3 were diagnosed with MDD and one with dysthymic disorder. Ninety families from the Amsterdam area participated. Sixty-three families contributed one patient and one sibling, 20 families contributed one patient and two siblings, three families contributed one patient and three siblings and one family contributed one patient and four siblings. Two families contributed two siblings, but no patients. One family contributed two controls. Fifty-three independent patients, 5 independent siblings and 81 independent controls participated, i.e. these individuals had no relatives in the sample.

#### Maastricht sample

The Maastricht sample consisted of 82 patients with psychotic disorder, 94 non-psychotic siblings of patients, and 78 controls. Of the patients, 59 were diagnosed with schizophrenia and 16 were diagnosed with schizoaffective disorder, 2 had a substance-induced psychotic disorder and 1 patient was diagnosed with brief psychotic disorder. The other patients (n = 4) were diagnosed with psychotic disorder not otherwise specified (NOS). In addition, 28 siblings and 16 controls had a diagnosis of MDD. A total of 50 families from Maastricht participated in the study. Thirty-one families contributed one patient and one sibling, seven families contributed one patient and two siblings, one family contributed one patient and three siblings and one family contributed two patients but no siblings. Four families contributed two siblings and one family contributed three siblings, but no patients. Five families contributed two controls. Forty-one independent patients, 35 independent siblings and 68 independent controls participated.

### Measures

#### Body mass index (BMI)

BMI was calculated as weight in kilograms divided by height in meters squared.

#### Positive and negative symptoms

Psychotic symptomatology was assessed with the Positive and Negative Syndrome Scale (PANSS) [36]. The scores of the individual items of the positive and negative symptom dimensions were summed.

#### Antipsychotic medication

Self-report present use and type of antipsychotic medication (AP) was documented. AP was categorized by generation, i.e. first generation antipsychotics (FGA) or second and third generation antipsychotics (SGA and TGA respectively). For the analyses, SGA and TGA were combined. In case of simultaneous use of an FGA and SGA or TGA, the AP type was classified as FGA.

### Serum sample acquisition and processing

Serum samples were acquired by venipuncture and were centrifuged and frozen within 24 hours. S100B was analyzed using a Liaison automated chemiluminescence analyzer according to the manufacturer's instructions (Diasorin). The lowest concentration of detection was 0.02 microgram per liter. ROC curves showed best accuracy for the Liaison Sangtec 100 assay [37]. All samples (from both locations) were analyzed at the same laboratory of the Radboud University Medical Centre, employing the same technique.

### Ethics statement

This study was approved by the standing ethics committees of the University Medical Centres of Utrecht (G.R.O.U.P. study), Amsterdam, Rotterdam and Maastricht. All subjects provided written informed consent in accordance with the committee's guidelines.

### Statistical analysis

The strategy was analysis in one sample followed by within-study replication in the other sample. Per sample, group differences in S100B were analyzed using multilevel random regression models because of hierarchical clustering occasioned by the fact that participants were clustered in families, compromising statistical independence of the observations [38]. This was done using the XTREG command in STATA (STATA corp, version 11). S100B was the dependent variable in the analyses and group (entered as both linear and dummy variables (controls = 0, siblings = 1 and patients = 2)) was the independent variable. Analyses were adjusted for the following a priori hypothesized confounders: sex, age and BMI. In addition, we tested whether S100B was not only conditional on group but also on sex, by examining group×sex interactions corrected for age and BMI. Interactions terms were evaluated by Wald test [39].

To account for the clinical heterogeneity in the patient samples, the association between group and S100B was examined in two subgroups: i) patients with a narrowly defined diagnosis of schizophrenia (i.e. excluding schizoaffective disorder, schizophreniform disorder, delusional disorder and psychotic disorder NOS), and ii) patients with any psychotic disorder with PANSS positive and negative scores of fifteen or higher.

As S100B may be elevated in mood disorders [40], sensitivity analyses were conducted, excluding controls and siblings with a history of affective disorders or other psychiatric morbidity.

Associations between the PANSS symptom levels and S100B were investigated in the patient samples using multilevel regression analyses for the Maastricht sample (given hierarchical clustering of patients within families) and multiple regression analyses for the Amsterdam sample, with S100B as the dependent variable and symptom scores as the independent variable. In order to visualize dose-response in case of an association, PANSS symptoms scores were entered as dummy variables representing the distribution of the scores divided by its tertiles. Analyses were corrected for sex and age.

Multilevel (Maastricht sample) and multiple regression procedures (Amsterdam sample) were also used to investigate associations between present use of AP (0 = no current use, 1 = current use) and S100B. Associations between type of AP and S100B were examined by comparing FGA and SGA/TGA to no AP (0 = no AP, 1 = FGA, 2 = SGA/TGA). AP analyses were corrected for sex, age and BMI.

## Results

### Descriptive analyses

Eighty-six percent of the Amsterdam patients and 77% of the Maastricht patients was male; for the control group these figures were 69% and 32% respectively. The mean age and BMI of all groups was higher in the Maastricht sample. Patients from Amsterdam had higher negative symptom levels than those from Maastricht. Symptom levels in siblings and controls were not reported because of the high proportion of missing values in the Amsterdam sample (Table 1).

**Table 1:** Demographic characteristics.

	Controls	Siblings	Patients
**Number of participants**			
Amsterdam	83	125	140
Maastricht	78	94	82
**Sex (male/female)**			
Amsterdam	57/26	57/68	120/20
Maastricht	25/53	44/50	63/19
**S100B (µg/l)**			
Amsterdam	0.070±0.028	0.070±0.029	0.075±0.033
Maastricht	0.076±0.028	0.078±0.039	0.083±0.078
**Age**			
Amsterdam	26.16±9.31	27.03±8.11	24.65±6.00
Maastricht	34.40±10.39	31.61±8.59	30.50±6.93
**BMI (kg/m^2^)**			
Amsterdam	22.41±2.47	22.94±3.37	23.88±3.69
Maastricht	24.39±4.74	24.80±4.56	25.96±4.13
**Positive symptoms (PANSS)**			
Amsterdam			12.00±5.18
Maatricht			11.50±5.25
**Negative symptoms (PANSS)**			
Amsterdam			16.21±6.83
Maastricht			11.17±4.66
**Present use of AP (yes/no)**			
Amsterdam			112/13 (15 NA)
Maastricht			72/10

± standard deviations reported. PANSS: Positive and negative syndrome scale; NA: not available. Means

Within the samples, patients and siblings were younger than controls in Maastricht while siblings were older than patients in Amsterdam. In Maastricht, BMI was higher in patients compared to controls, but not siblings. Patients from Amsterdam had a higher BMI compared to both controls and siblings (Table 1).

Out of 140 patients from the Amsterdam sample, 112 reported the present use of an AP. Thirty-five patients used olanzapine, 31 used risperidone, 13 used clozapine, 8 used aripiprazole, 5 quetiapine, 5 haloperidol and 2 penfluridol. Two used flupenthixol, one used pimozide, and one sulpiride. Three individuals used a combination of haloperidol and clozapine, 2 used risperidone and quetiapine, 1 a combination of aripiprazole and olanzapine and 1 person used pimozide and risperidone. Two patients were unable to provide the name of the current AP. For fifteen patients, AP data was missing.

In the Maastricht sample, 72 out of 82 patients currently used AP medication. Sixteen patients used risperidone, 16 used olanzapine, 12 used aripiprazole, 7 used clozapine, 5 quetiapine, 3 haloperidol and 2 used amisulpride. Two patients used zuclopenthixol and bromperidol, respectively. Two persons used a combination of clozapine and aripiprazole, 1 a combination of aripiprazole and olanzapine, 1 haloperidol and pipamperone, 1 zuclopenthixol and quetiapine, 1 clozapine and clotiapine, 1 risperidone and aripiprazole, 1 risperidone and paliperidone, and 1 a combination of risperidone, amisulpride and clotiapine. One sibling with a diagnosis of depression used a low dose of olanzapine.

### Associations between group and S100B

Although the direction of the effect was positive, there was no significant association between group (linear trend) and S100B in the samples (Amsterdam: B = 0.002, 95% CI −0.002 to 0.006, p = 0.377; and Maastricht: B = 0.004, 95% CI −0.006 to 0.014, p = 0.407).

Between group (dummy variable) comparisons showed that S100B levels in patients and siblings were not significantly different from controls (Amsterdam: P vs. C: B = 0.004, 95% CI −0.005 to 0.013, p = 0.351; S vs. C: B = 0.000, 95% CI −0.009 to 0.008, p = 0.926; and Maastricht: P vs. C: B = 0.008, 95% CI −0.011 to 0.028, p = 0.410; S vs. C: B = 0.002, 95% CI −0.016 to 0.021, p = 0.797), nor was there a large or significant difference between patients and siblings (Table 2).

**Table 2:** Associations between group and S100B.

	S vs. C	P vs. C	P vs. S
	B (95% CI), p-value	B (95% CI), p-value	B (95% CI), p-value
**Group (AD +)**			
Amsterdam	0.000 (−0.009–0.008), p = 0.926	0.004 (−0.005–0.013), p = 0.351	0.005 (−0.003–0.012), p = 0.207
Maastricht	0.002 (−0.016–0.021), p = 0.797	0.008 (−0.011–0.028), p = 0.410	0.006 (−0.011–0.023), p = 0.504
**Group (AD −)**			
Amsterdam	0.000 (−0.009–0.009), p = 0.995	0.004 (−0.005–0.013), p = 0.396	0.004 (−0.003–0.011), p = 0.254
Maastricht	0.001 (−0.020–0.023), p = 0.897	0.009 (−0.013–0.030), p = 0.436	0.007 (−0.013–0.027), p = 0.482
**Group (SCZ only)**			
Amsterdam	−0.001 (−0.010–0.008), p = 0.821	0.003 (−0.006–0.013), p = 0.473	0.004 (−0.003–0.012), p = 0.268
Maastricht	0.001 (−0.014–0.016), p = 0.875	−0.001 (−0.019–0.017), p = 0.926	−0.002 (−0.017–0.013), p = 0.787
**Group (PANSS pos≥15)**			
Amsterdam	−0.002 (−0.011–0.006), p = 0.598	0.008 (−0.003–0.020), p = 0.165	0.010 (0.000–0.020), p = 0.040
Maastricht	0.003 (−0.010–0.015), p = 0.702	−0.005 (−0.026–0.017), p = 0.683	−0.007 (−0.027–0.013), p = 0.487
**Group (PANSS neg≥15)**			
Amsterdam	−0.001 (−0.010–0.008), p = 0.850	0.010 (0.000–0.020), p = 0.051	0.011 (0.002–0.020), p = 0.016
Maastricht	0.002 (−0.011–0.015), p = 0.719	0.011 (−0.012–0.034), p = 0.344	0.009 (−0.012–0.029), p = 0.410

% CI: 95% confidence interval. B represents the regression coefficient of the multilevel regression analyses. 95

S vs. C: sibling versus control; P vs. C: patient versus control; P vs. S: patient versus sibling.

+: affective disorders in controls and siblings included; AD −: affective disorders in controls and siblings excluded; SCZ: schizophrenia only in patient groups; PANSS: Positive and negative syndrome scale; PANSS pos≥15: PANSS positive symptom level above or equal to 15 in patient groups; PANSS neg≥15: PANSS negative symptom level above or equal to 15 in patient groups. AD

### Association between group and S100B: subgroup analyses

#### Narrow schizophrenia diagnosis only

Excluding patients with a diagnosis other than schizophrenia altered the pattern of effect sizes in some instances, but none of the between-group comparisons was statistically significant (Table 2), nor the effect of group as a linear trend variable (Amsterdam: B = 0.001, 95% CI −0.004 to 0.006, p = 0.696; and Maastricht: B = 0.001, 95% CI −0.008 to 0.010, p = 0.806).

#### All psychotic disorders with PANSS scores ≥15 (on positive and negative subscales respectively)

At a cut-off of fifteen in PANSS positive symptom levels, 33 patients remained in the Amsterdam sample and 14 remained in the Maastricht sample. There were no significant associations between S100B and group (linear trend) (Amsterdam: B = 0.002, 95% CI −0.004 to 0.008, p = 0.539; and Maastricht: B = 0.000, 95% CI −0.010 to 0.010, p = 0.966). In the between-group comparison, patients had significantly higher S100B levels compared to siblings in the Amsterdam sample, although patients and siblings were not significantly different from controls. In the Maastricht sample, there were no large or significant associations between group and S100B, with an opposite direction of effect sizes (Table 2).

At a cut-off of fifteen in PANSS negative symptom levels, 72 Amsterdam and 13 Maastricht patients remained in the analyses. There was no significant association between S100B and group as a linear trend variable (Amsterdam: B = 0.005, 95% CI 0.000 to 0.010, p = 0.076; and Maastricht: B = 0.005, 95% CI −0.005 to 0.015, p = 0.349). However, between-group comparisons showed that patients in Amsterdam had significantly higher S100B levels than siblings (B = 0.011, 95% CI 0.002 to 0.020, p = 0.016), and controls - at trend-level (B = 0.010, 95% CI 0.000 to 0.020, p = 0.051). This was not replicated in the Maastricht sample (Table 2).

#### Exclusion of affective disorders and other psychiatric morbidity in siblings and controls

The exclusion of controls and siblings with a history of affective disorder or other psychiatric morbidity did not affect the findings when using the original patient samples, or any of the above described sub-analyses (Table 2).

### Associations between symptomatology and S100B

In patients (all diagnoses), negative symptoms were positively associated with S100B in the Amsterdam sample (B = 0.001, 95% CI 0.000 to 0.002, p = 0.005), but not in the Maastricht sample (B = 0.002, 95% CI −0.002 to 0.005, p = 0.308). In the Amsterdam sample, progressively higher negative symptom levels were associated with progressively higher S100B levels, although only the highest negative symptom level reached statistical significance (high vs. low symptom group: B = 0.016, 95% CI 0.003 to 0.030, p = 0.020; intermediate vs. low symptom group: B = 0.010, 95% CI −0.003 to 0.024, p = 0.127).

There was no large or statistically significant association between positive symptoms and S100B in the Amsterdam or Maastricht sample (B = 0.001, 95% CI 0.000 to 0.002, p = 0.098; and B = −0.001, 95% CI −0.004 to 0.002, p = 0.483 respectively).

### Associations between antipsychotic medication and S100B

In patients, the present use of AP medication was not significantly associated with S100B (Amsterdam: B = −0.003, 95% CI −0.022 to 0.017, p = 0.781; Maastricht: B = 0.027, 95% CI −0.024 to 0.078, p = 0.302). Similarly, there was no effect of type of AP, comparing FGA and SGA/TGA users to AP-free patients and comparing FGA to SGA/TGA users (Table 3).

**Table 3:** Associations between AP medication and S100B.

	FGA vs. no AP	SGA/TGA vs. no AP	SGA/TGA vs. FGA
**Patients**			
Amsterdam	0.005 (−0.020–0.029), p = 0.713	−0.004 (−0.024–0.015), p = 0.659	−0.009 (−0.028–0.010), p = 0.335
Maastricht	−0.001 (−0.076–0.073), p = 0.970	0.030 (−0.021–0.081), p = 0.248	0.031 (−0.029–0.092), p = 0.309

% confidence interval) and p-value reported. B represents the regression coefficient of the regression analyses. B (95

AP: antipsychotic medication; FGA: first generation AP; SGA/TGA: second and third generation AP.

### Group×sex interactions and S100B

There was no evidence for significant group×sex interactions in either sample (Amsterdam: χ^2^ = 0.35, p = 0.839; Maastricht: χ^2^ = 1.45, p = 0.484).

## Discussion

Contrary to the hypothesis, there was no statistically significant association between group and S100B. Serum S100B levels in patients and siblings were not different from controls. In the Amsterdam sample, there was a significant positive association between negative symptoms and S100B, which was not replicated in the Maastricht sample. There was no large or significant association between S100B and positive symptoms or the present use or type of AP in either sample.

### Findings

#### (Familial) risk of psychotic disorder and S100B

We did not detect significantly elevated S100B in patients, in disagreement with a range of previous small studies. Other investigations suggesting an absence of elevated S100B in schizophrenia are limited. One study found unaltered S100B levels in unmedicated patients compared to controls, although S100B was elevated in patients who had been treated with AP for 3 weeks compared to unmedicated patients and controls [10]. In another study, a small sample of patients with schizophrenia had higher levels of S100B in cerebrospinal fluid (CSF) but not serum (after Bonferroni correction) [11]. There is one study reporting reduced S100B levels [12]. Although the absence of elevated S100B in patients does not agree with most literature, the current results provided a replication in two samples that were considerably larger than earlier work (except for two studies [18], [41]). Another difference between the current and other studies concerns the patient population. The current samples comprised mainly outpatients, generally not in an acute stage of the disorder, with a mean illness duration of <10 years, whereas the majority of previous work was conducted in hospitalized patients. The evidence to date may thus be suggestive of S100B elevation in inpatient populations with presumably more severe symptomatology. Furthermore, a considerable proportion of the patients in the current study had a psychotic disorder other than schizophrenia, while previous studies were conducted specifically in schizophrenic patients. However, the diagnosis of schizophrenia does not refer to a natural illness type. The different diagnostic categories in the DSM-IV chapter of psychotic disorders likely reflect phenotypic diversity related to a shared liability. In addition, the sensitivity analyses (excluding patients with a diagnosis of psychotic disorder other than schizophrenia) did not provide evidence for differences between groups. It should further be noted that serological and histological S100B alterations have been associated with affective disorders [40], [42]. In the current sample in- or exclusion of all psychiatric morbidity in siblings and controls yielded the same results: the absence of association between serum S100B and risk of psychotic disorder.

This is the first study to investigate serum S100B as a marker of familial risk of psychotic disorder. The use of non-psychotic siblings in the study design makes it possible to establish the effect of shared familial risk of psychotic disorder, without confounding by disease-related factors. The data did not suggest that serum S100B is an intermediate phenotype for psychotic disorder. The absence of S100B alterations in our relatively stable patient and non-psychotic sibling populations, in combination with the previously reported increased S100B levels in hospitalized patient populations, may suggest that S100B elevations reflect fluctuating disease processes in certain patient populations, i.e. that they are related to the clinical “state”. This reasoning may be underscored by the (albeit weak) association between S100B and negative symptoms in the Amsterdam sample. Alternatively, it may be that previous work reporting positive associations was influenced by publication/reporting bias, particularly given the suggestion of excess significance bias in the literature, affecting the representation of true associations between psychiatric phenotypes and biological measures [43].

#### Symptomatology and S100B

Narrowing down the diagnosis of schizophrenia did not affect the pattern of findings. In addition, patients in the Amsterdam sample with more severe positive symptomatology (PANSS scores ≥15) had higher S100B levels compared to siblings but not compared to controls, while patients with more severe negative symptoms had higher S100B levels compared to both siblings and controls (at trend-level), regardless of diagnosis. This was not replicated in the Maastricht sample. A cut-off of 15 for the PANSS positive and negative symptom levels is clinically quite conservative. A higher cut-off, however, would have further reduced the samples size. These findings suggest that the absence of S100B elevation was not due to diagnostic heterogeneity.

To examine whether S100B elevation may be associated with higher symptom levels in patient populations, main effects of symptomatology on S100B were assessed. The positive association between negative symptoms and S100B, as found in the Amsterdam sample, has been reported previously [10], [13], [16], [17]. Despite the statistical significance of this finding, the clinical relevance is debatable given the small effect size. Furthermore, the association between negative symptoms and S100B levels was not replicated in the Maastricht sample, although size and direction of the association were similar. Patients in Amsterdam had higher negative symptom levels than patients in Maastricht, possibly due to the higher proportion of men in the Amsterdam sample. The negative symptom level in the Maastricht patient sample was also quite low compared to other studies that did find an association using the PANSS.

A recent review [44] expressed the opinion that S100B studies have been tainted by bias due to selection of patients with high negative symptom levels. Nevertheless, certain explanations for a true positive association between S100B and negative symptoms could be conceived. First, neuroimaging work [45] has confirmed white matter tracts as sites of profuse S100B expression (by glia and oligodendrocytes) and white matter abnormalities have been associated with negative symptoms [46]–[50]. Second, S100B may reflect a compensatory mechanism instead of a causative process, cueing in recovery at a stage of illness when positive symptoms have begun to remit and negative symptoms become more prominent.

#### AP medication and S100B

Previously reported effects of AP on S100B have been inconsistent. A decline in S100B after several weeks of AP treatment has been reported [10], [17], and in vitro work by Steiner [51] also suggests that S100B levels are normalized by AP. However, a longitudinal study showed that S100B remained increased after 24 weeks of treatment [18]. In other studies, medication-free patients with schizophrenia had higher serum S100B than healthy controls [52] and drug-naïve first-episode patients had significantly higher serum S100B levels than chronic patients on long-term AP [41], with the latter group showing S100B elevation compared to healthy controls. These studies show that S100B elevation cannot be ascribed to AP. A recent meta-analysis did not find an association between S100B in schizophrenia and AP treatment [9], in line with the current findings.

### Methodological considerations

The strength of the present study was that it comprised two large patient-sibling-control samples, allowing for independent replication. As an additional validation, we repeated the analyses combining the Amsterdam and Maastricht samples to increase power. In the combined sample, the absence of a significant positive association between S100B and group was upheld. The association between negative symptoms and S100B in patients (which was only present in the Amsterdam sample) was not significant (p = 0.081) in the combined sample (results available on request).

Certain limitations should be addressed. There is debate about the specificity of S100B as a marker of CNS pathology as S100B is expressed in other cells and tissues in physiological circumstances, e.g. adipocytes, chondrocytes, lymphocytes and melanocytes [5], [6], [53], as well as in pathological conditions, e.g. cardiomyocytes after infarction [5], [6], [54]. One way to circumvent this issue is to obtain S100B levels in the CSF. In a comparative serum/CSF study, serum S100B was found to mirror CSF concentrations [13]. Corroborating evidence comes from a study reporting that extracerebral sources of S100B did not alter S100B serum levels [55]. Thus, despite the diverse origins of serum S100B, evidence suggests that serum S100B is correlated with CSF S100B.

As Steiner and colleagues found that S100B is associated with BMI, we adjusted for BMI [53]. In contrast, Pham and colleagues did not find an association between BMI and S100B [55]. The pattern of our findings was unaltered when BMI was removed as a covariate from the analysis. To clarify a potential link to insulin resistance [20], [56], it may be useful to measure HbA1c in future studies.

## Conclusions

Our findings suggest the following: i) S100B is not an intermediate phenotype of psychotic disorder; and ii) S100B is not elevated in the “general population” of individuals with psychotic disorder.
